# Electromyography Data Processing Impacts Muscle Synergies during Gait for Unimpaired Children and Children with Cerebral Palsy

**DOI:** 10.3389/fncom.2017.00050

**Published:** 2017-06-06

**Authors:** Benjamin R. Shuman, Michael H. Schwartz, Katherine M. Steele

**Affiliations:** ^1^Department of Mechanical Engineering, University of WashingtonSeattle, WA, United States; ^2^WRF Institute for Neuroengineering, University of WashingtonSeattle, WA, United States; ^3^James R. Gage Center for Gait and Motion Analysis, Gillette Children's Specialty HealthcareSt. Paul, MN, United States; ^4^Department of Biomedical Engineering, University of MinnesotaMinneapolis, MN, United States

**Keywords:** electromyography, muscle synergies, low pass filtering, amplitude scaling, walk-DMC, non-negative matrix factorization, cerebral palsy

## Abstract

Muscle synergies calculated from electromyography (EMG) data identify weighted groups of muscles activated together during functional tasks. Research has shown that fewer synergies are required to describe EMG data of individuals with neurologic impairments. When considering potential clinical applications of synergies, understanding how EMG data processing impacts results and clinical interpretation is important. The aim of this study was to evaluate how EMG signal processing impacts synergy outputs during gait. We evaluated the impacts of two common processing steps for synergy analyses: low pass (LP) filtering and unit variance scaling. We evaluated EMG data collected during barefoot walking from five muscles of 113 children with cerebral palsy (CP) and 73 typically-developing (TD) children. We applied LP filters to the EMG data with cutoff frequencies ranging from 4 to 40 Hz (reflecting the range reported in prior synergy research). We also evaluated the impact of normalizing EMG amplitude by unit variance. We found that the total variance accounted for (tVAF) by a given number of synergies was sensitive to LP filter choice and decreased in both TD and CP groups with increasing LP cutoff frequency (e.g., 9.3 percentage points change for one synergy between 4 and 40 Hz). This change in tVAF can alter the number of synergies selected for further analyses. Normalizing tVAF to a z-score (e.g., dynamic motor control index during walking, walk-DMC) reduced sensitivity to LP cutoff. Unit variance scaling caused comparatively small changes in tVAF. Synergy weights and activations were impacted less than tVAF by LP filter choice and unit variance normalization. These results demonstrate that EMG signal processing methods impact outputs of synergy analysis and z-score based measures can assist in reporting and comparing results across studies and clinical centers.

## Introduction

Muscle synergies have been used to describe the low-dimensional sets of weighted muscle groups that are recruited during functional tasks (Tresch and Jarc, 2009; Bizzi and Cheung, 2013). Prior research has theorized that the nervous system uses these synergies as a simplified method of control, rather than controlling each muscle individually. Recent research has applied muscle synergies as a framework to evaluate altered neuromuscular control in individuals with neurologic disorders. Research on individuals with stroke and CP have shown that fewer synergies are required to describe EMG data during functional tasks compared to unimpaired individuals, and this reduction in activation complexity may contribute to movement impairments (Cheung et al., 2009b; Clark et al., 2010; Monaco et al., 2010; Allen et al., 2013; Roh et al., 2013; Routson et al., 2014; Steele et al., 2015). However, despite the general agreement that synergy complexity is reduced in stroke and CP, there is no consistent methodology for calculating muscle synergies. Prior to calculating muscle synergies, raw EMG data are processed to generate linear envelopes describing the activation of each muscle during a task such as walking. In general, this process consists of an initial filtering (e.g., high pass or band pass filtering), full wave rectification, low pass (LP) filtering, and amplitude scaling. As researchers investigate potential clinical applications of synergy analyses, such as in clinical gait analysis, understanding the impact of EMG preprocessing is important to compare across studies or clinical centers.

Prior synergy research has used a wide variety of EMG preprocessing methods. In particular, a wide range of LP filters have been used to smooth EMG data, with LP cutoff frequencies ranging from 1 (Muceli et al., 2010) to 40 Hz (Torres-Oviedo and Ting, 2010), and including many intermediate values including 4 (Clark et al., 2010), 10 (Steele et al., 2015), 20 (Cheung et al., 2009a), 30 (Torres-Oviedo et al., 2006), or 35 Hz (Sawers et al., 2015). Despite this, there has been little research examining how muscle synergy calculations are impacted by these LP filter choices. Kleissen (1990) showed large differences in smoothness and cycle-to-cycle variability in EMG envelopes from the gluteus medius during gait when LP filtered at 3.4 or 25 Hz. For synergies, Van der Krogt et al. (2016) showed that the total variance accounted for (tVAF) by a given number of synergies was reduced with increasing LP cutoff frequency in children with CP for EMG data LP filtered between 2 and 25 Hz. Since tVAF is commonly used to pick or choose the number of synergies for further analysis (e.g., the number of synergies required for tVAF > 90 or 95%), impacts of LP filtering on tVAF can further impact conclusions about muscles that are activated together or differences in synergies between control and clinical populations. Hug et al. (2012) noted that the number of synergies required to explain 90% of the variance in EMG data changed between 4, 10 and 15 Hz LP filters. However, it has not been shown how LP filters can affect calculated synergy weights, which describe muscles commonly activated together, or synergy activation curves, which describe how each synergy is activated over time.

After filtering, the processed EMG data amplitudes are often scaled through one of several methods. These include peak measured amplitude (Clark et al., 2010; Steele et al., 2015), maximum voluntary contractions (Berger et al., 2013; Zelik et al., 2014), or median trial maximums (Cheung et al., 2009b). Additionally, for synergy analyses, prior research has scaled the amplitude so that each muscle has unit variance (Torres-Oviedo et al., 2006; Roh et al., 2013; Sawers et al., 2015). Unit variance scaling has been applied to avoid larger representations of high-variance muscles in the output synergy weights (Cheung et al., 2009a). As with filter cutoff, the effects of amplitude scaling on synergy outputs remains unclear.

To reduce potential impacts of EMG preprocessing on synergy results and facilitate comparison across studies or clinical centers, some prior research has suggested normalizing data to a z-score. For example, the dynamic motor control index during walking (walk-DMC) provides a summary measure of synergy complexity by normalizing tVAF by one synergy to the average and standard deviation of a group of unimpaired individuals (Steele et al., 2015; Schwartz et al., 2016). By normalizing to a group of controls from a given clinic or research lab, walk-DMC may help to reduce the impacts of different equipment, muscles, or EMG preprocessing methods across institutions. walk-DMC differs between typically-developing (TD) children and children with CP and is associated with treatment outcomes for children with CP (Schwartz et al., 2016). The impact of EMG processing on walk-DMC has not been investigated.

The goal of this research was to examine how EMG processing, specifically the choice of LP filter cutoff frequency and amplitude scaling, affects synergy analyses for TD children and children with CP. We evaluated how processing choices impact synergy complexity, in terms of tVAF and walk-DMC. We also evaluated how synergy weights and synergy activation curves change with processing choices. We hypothesized that walk-DMC would be more consistent across EMG processing conditions than tVAF, and that synergy weights and activations would change across processing parameters. Further, we hypothesized that both the TD and CP children's synergies would be similarly impacted by processing choices. Understanding the impact of EMG data processing on synergy outputs will help inform comparisons between studies and guide future clinical applications of synergy analyses.

## Methods

Human subjects' approval was obtained from both the University of Washington and the University of Minnesota for this study.

We retrospectively analyzed individuals who previously received gait analysis at Gillette Children's Specialty Healthcare. For this study, we sought to identify 40 individuals with diplegic CP, belonging to each of the Gross Motor Function Classification System (GMFCS) Levels I, II, and III (120 total participants), who had EMG data collected from five muscles during routine clinical gait analysis. For GMFCS Level III, only 33 individuals met these inclusion criteria. Data for TD children were obtained from the control database at Gillette Children's Specialty Healthcare. Table 1 summarizes the demographic data for all participants in this study.

**Table 1:** Study population.

	**N**	**Sex F:M**	**Age (year)**	**Height (m)**	**Mass (kg)**
TD	73	30:43	10.5 ± 3.5	1.44 ± 0.20	40.3 ± 13.3
GMFCS I	40	22:18	10.4 ± 4.8	1.35 ± 0.19	33.6 ± 15.6
GMFCS II	40	14:26	10.9 ± 5.8	1.34 ± 0.22	33.5 ± 15.9
GMFCS III	33	17:16	12.2 ± 9.4	1.28 ± 0.18	32.8 ± 21.9

*N, number of participants; F, Female; M, Male; GMFCS, Gross Motor Function Classification System*.

### Electromyography data

Surface EMG data (Motion Laboratory Systems, Baton Rouge, LA, USA) were collected at 1,080 Hz for five muscles (rectus femoris, medial hamstrings, lateral hamstrings, medial gastrocnemius, and tibialis anterior) during barefoot walking at a self-selected speed. For each individual, one limb was randomly selected for analysis. We took the middle 80% of the entire gait trial to avoid transient accelerations and decelerations near the beginning and end of the trial and maximize data for analysis (Oliveira et al., 2014). Raw EMG data were band pass filtered between 35 and 500 Hz upon collection.

**Figure 1:**
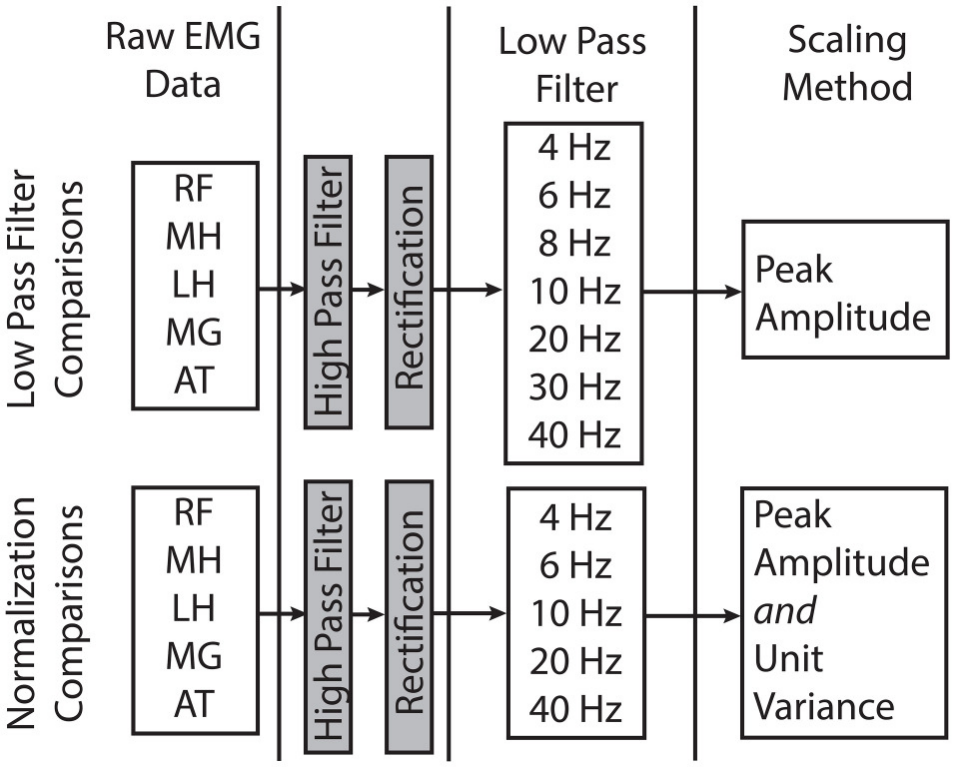
Processing steps and filter parameters used in this study to evaluate the impact of LP filter choice and amplitude normalization methods on the results of synergy analyses. RF, rectus femoris; MH, medial hamstrings; LH, lateral hamstrings; MG, medial gastrocnemius; AT, anterior tibialis.

EMG data for each child were digitally processed with a high pass (HP) filter and a set of varying LP filters (Figure 1). The filter parameters were based upon prior studies of synergies during gait (Torres-Oviedo et al., 2006; Cheung et al., 2009a; Clark et al., 2010; Torres-Oviedo and Ting, 2010; Steele et al., 2015). The pipeline for processing EMG data for synergy analysis involves the following sequence: (1) HP filtering (40 Hz) to eliminate DC drift and movement artifacts, (2) full wave rectification, and (3) LP filtering to create a linear envelope of muscle activity. For both filtering steps, we used 4th order Butterworth filters, which have commonly been used in synergy analyses (Neptune et al., 2009; Clark et al., 2010; Allen et al., 2013; Routson et al., 2014). The specific LP cutoff frequencies evaluated were: 4, 6, 8, 10, 20, 30, and 40 Hz. Since maximum voluntary contractions are not collected as part of clinical care at Gillette, EMG data were scaled to the peak amplitude for each muscle. Since some prior synergy analyses scale EMG data to unit variance, we also investigated the impact of unit variance scaling with varying filter parameters. Each EMG channel was scaled to unit variance across the walking trial. After filtering and amplitude scaling, the EMG envelopes were down-sampled to 100 Hz to reduce synergy computation time.

### Synergy analysis

**Figure 2:**
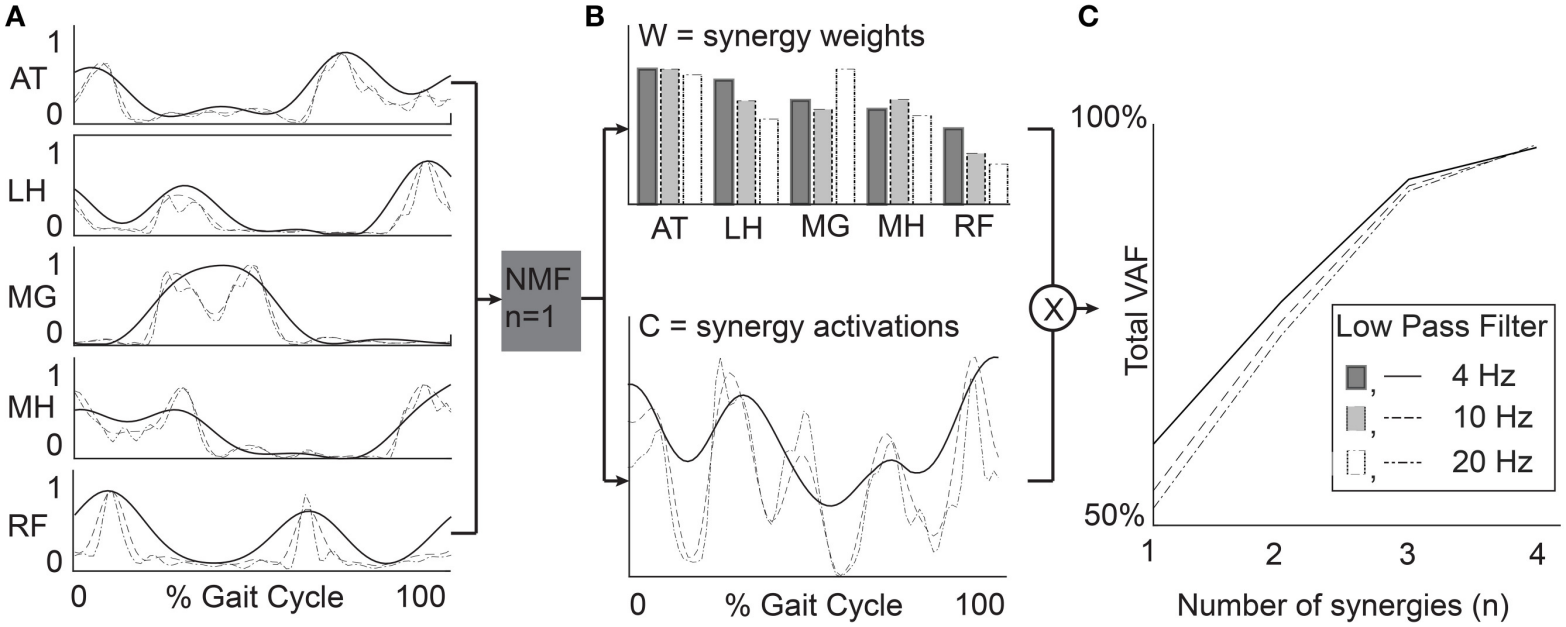
Example data from a representative TD participant. **(A)** EMG data were processed with varying LP filter cutoffs. **(B)** Synergy weights (*W*) and activations (*C*) were calculated for *n* = 1–4 synergies. **(C)** Total variance accounted for by *n* synergies provides a measure of synergy complexity and is often used to select a number of synergies for further analysis (e.g., tVAF > 90%). RF, rectus femoris; MH, medial hamstrings; LH, lateral hamstrings; MG, medial gastrocnemius; AT, anterior tibialis.

Synergies were calculated from the EMG data processed with each filtering condition using non-negative matrix factorization (NMF) (Figure 2). This method calculates a set of synergy weights (*W*_*mxn*_) and synergy activations (*C*_*nxt*_), such that *EMG* = *W* × *C*+*error* where *n* is the number of synergies (1–4 in this study), *m* is the number of muscles (5 in this study), and *t* is equal to the number of EMG data points. The error term is defined as the difference between the filtered EMG data and the EMG data reconstructed from the product of the synergy weights and activations. We calculated synergies with NMF in Matlab (Statistics and Machine Learning Toolbox, MathWorks, Inc., Natick, Massachusetts, United States) using the following parameters: 50 replicates, 1,000 maximum iterations, a 1 × 10^−4^ minimum threshold for convergence, and a 1 × 10^−6^ threshold for completion. Note that specific synergies were calculated separately for each number of synergies specified. In other words, a synergy from a 2-synergy solution may be different than all of the synergies from a 3-synergy solution.

We used three measures to evaluate synergy complexity: (1) the total variance accounted for (tVAF), (2) the number of synergies required for tVAF > 90%, and (3) a z-score of tVAF (walk-DMC). The tVAF by n synergies was defined as one minus the ratio of the sum of squared errors to the sum of filtered EMG data over all muscles (Equation 1, Ting and Macpherson, 2005). Traditionally, tVAF is used to define the number of synergies to evaluate in synergy analyses. For each LP filter, we used a *t*-test to compare tVAF and a Mann-Whitney *U*-test to compare the number of synergies between CP and TD groups.

(1) $$\begin{array}{l} tVAF_{n}=\left(1-\frac{\left[\sum_{j}^{t}\sum_{i}^{m}{\left(error\right)}^{2}\right]}{\left[\sum_{j}^{t}\sum_{i}^{m}{\left(EMG\right)}^{2}\right]}\right)\times 100\% \end{array}$$

walk-DMC is a z-score based upon tVAF by one synergy (tVAF_1_, Equation 2), and uses the average and standard deviation of tVAF_1_ (*tVAF*_*AVG*_ and *tVAF*_*SD*_) from unimpaired controls. Thus, the average walk-DMC score for the TD group is 100, and each 10-point deviation represents one standard deviation from the TD controls. Note that a higher tVAF_1_ results in a lower walk-DMC score. For example, a walk-DMC of 80 indicates that an individual's tVAF_1_ during walking is two standard deviations above the TD group, suggesting simplified control.

(2) $$\begin{array}{l} walk-DMC=100+10\left[\frac{tVAF_{AVG}-tVAF_{1}}{tVAF_{SD}}\right] \end{array}$$

To evaluate the effect of filter parameters on synergy weights and activations, we calculated the correlation coefficients comparing synergy weights and activations across all filter conditions. We computed the average correlation coefficients between the *W* matrices output by NMF for each of the LP filtering conditions. Similarly, we computed the average correlation coefficients for synergy activations between the *C* matrices output by NMF from each of the LP filtering conditions.

Since some prior studies (Torres-Oviedo et al., 2006; Cheung et al., 2009a) scale EMG data for each muscle to unit variance before running NMF, we also evaluated the impact of unit variance scaling on the resulting synergies. We compared the outputs of synergy analyses performed with EMG scaled by unit variance and by peak activation. We calculated the change in average tVAF_1_ and walk-DMC with each LP filter condition to examine the impact of unit variance scaling on synergy complexity. Similarly, we calculated the correlation coefficients in synergy weights (*W*) and activations (*C*) with each LP filter condition between the unit variance and peak activation scaling methods. Note that EMG may be scaled directly to unit variance (Cheung et al., 2009a; Roh et al., 2013) or scaled to peak amplitude and then to unit variance (Torres-Oviedo et al., 2006; Hayes et al., 2014; Sawers et al., 2015) with equivalent synergy outputs. In this paper, we first scaled to peak amplitude and then to unit variance.

## Results

### Synergy complexity

**Figure 3:**
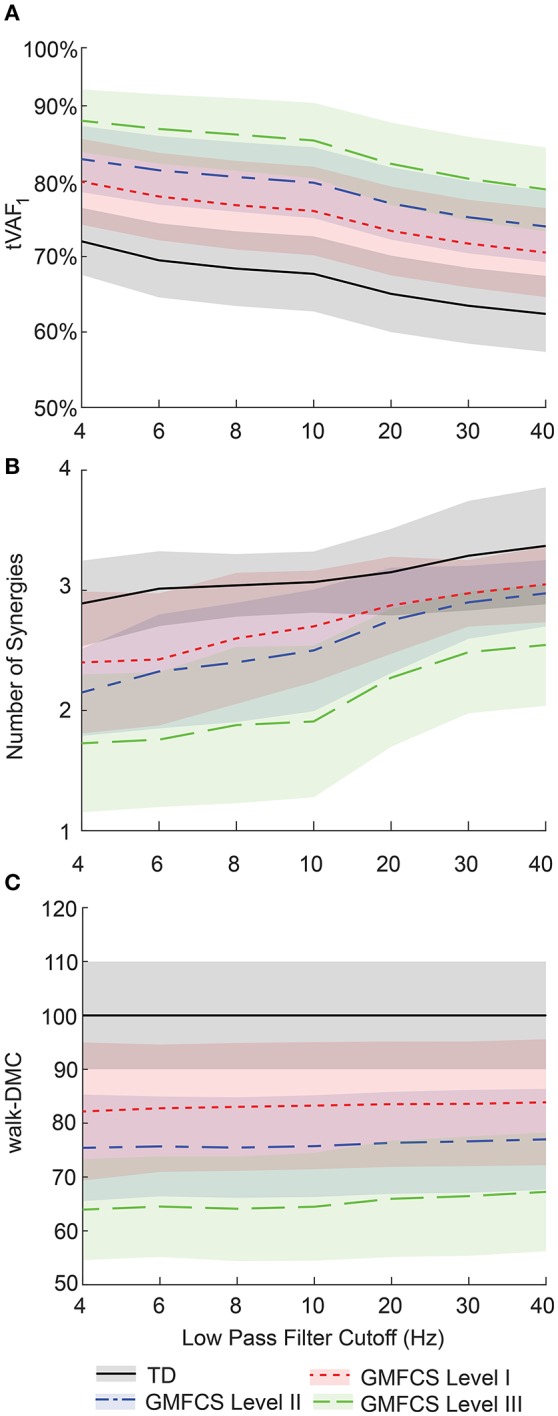
Average ± one standard deviation of **(A)** total variance accounted for by one synergy (tVAF_1_), **(B)** number of synergies for tVAF > 90%, and **(C)** walk-DMC across LP cutoff frequencies for TD and CP groups. As LP filter cutoff frequency increased, tVAF decreased and number of synergies increased for all groups. Average walk-DMC scores had minimal changes across LP cutoff frequencies.

LP filter cutoff frequency impacted synergy complexity, as measured by tVAF. Varying the LP cutoff frequency from 4 to 40 Hz decreased tVAF_1_ by 9.6 percentage points (i.e., from 72.0 to 62.4%) for the TD group, and 9.4, 8.9, and 9.1 percentage points for the GMFCS Level I, II, and III groups, respectively (Figure 3A). For individual participants, changes in tVAF_1_ ranged from 2.2 to 15.1 percentage points across LP filtering conditions. For more than one synergy, tVAF_2−4_ also decreased with increasing LP cutoff frequency with an average absolute reduction in tVAF of 6.5 percentage points for tVAF_2_, 3.8 percentage points for tVAF_3_, and 1.6 percentage points for tVAF_4_, across all participants. Despite changes in tVAF_*n*_ with LP cutoff frequency, tVAF was still significantly greater in CP compared to TD across all LP cutoff frequencies and numbers of synergies (*t*-test, *p* < 0.01 for all comparisons).

**Figure 4:**
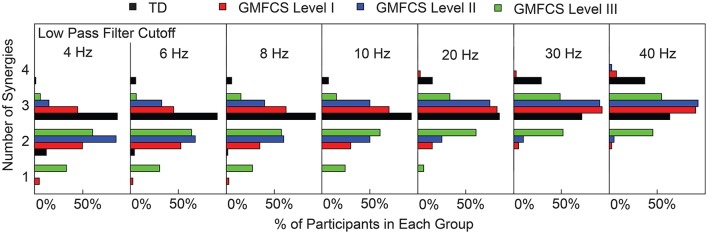
Number of synergies required for tVAF > 90%. Each TD and CP group is shown as the percentage of the total number of individuals in that group. As LP cutoff frequency increased the number of synergies increased for all groups.

Changes in tVAF influenced the choice of number of synergies (Figure 3B). When we applied a threshold of tVAF > 90% to identify the number of synergies, only 27% of individuals with CP and 52% of TD had the same the number of synergies across all LP cutoff frequencies (Figure 4). The average number of synergies during walking with the 90% tVAF threshold was 2.12 (0.58) and 2.89 (0.36) for CP and TD groups when we applied a 4 Hz LP cutoff frequency, vs. 2.88 (0.43) and 3.37 (0.49) with a 40 Hz LP cutoff frequency. However, the number of synergies was significantly less in CP compared to TD across all LP cutoff frequencies (Mann-Whitney *U*-test, *p* < 0.01 for all cutoff frequencies). A total of 69% of children with CP and 44% of the TD group increased the number of synergies by one with increasing LP cutoff frequency, while 4% increased by two synergies in both groups. Increasing LP cutoff frequency did not always lead to a greater number of synergies; four TD children decreased the number of synergies with increasing LP cutoff frequency.

Walk-DMC reduced the impact of LP cutoff frequency on synergy complexity. Between 4 and 40 Hz, GMFCS Levels I, II, and III average walk-DMC scores increased by 1.7, 1.6, and 3.3 points, respectively (Figure 3C). Since walk-DMC normalizes tVAF_1_ based upon the mean and standard deviation of the TD group, the TD group's average walk-DMC does not change (average of 100 with a 10 point standard deviation). For individual participants, the change in walk-DMC with LP cutoff frequency ranged from <0.01 to 15.4 points with an average change of 4.0 points. Some individual's walk-DMC increased with increasing LP cutoff frequency, while others decreased.

### Synergy weights

**Figure 5:**
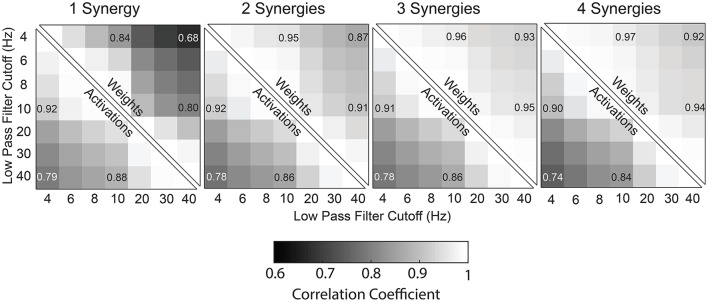
Correlation coefficients of synergy weights and synergy activations between LP cutoff frequencies, averaged across all subjects (TD and CP) for one to four synergies.

Similar to tVAF, changes in LP cutoff frequency also impacted synergy weights. Synergy weights calculated with a 4 and 40 Hz LP filter had an average correlation coefficients of 0.68, 0.87, 0.93, and 0.92 for 1–4 synergies, respectively (Figure 5). The average correlation coefficients by group were 0.68, 0.89, 0.94, and 0.93 for the TD children for 1–4 synergies, respectively; 0.69, 0.86, 0.94, and 0.92 for GMFCS Level I; 0.63, 0.85, 0.92, and 0.91 for GMFCS Level II; and 0.68, 0.86, 0.90, and 0.90 for GMFCS Level III. For an individual participant, the minimum correlation coefficient of synergy weights across LP cutoff frequencies was <0.01, 0.09, 0.47, and 0.57 for 1–4 synergies. Between 4 and 40 Hz, the correlation coefficient was >0.8 for 56, 20, 11, and 17% of all individuals for 1–4 synergies.

### Synergy activations

Synergy activations calculated with 4 or 40 Hz LP filters had an average correlation coefficient of 0.79, 0.78, 0.78, and 0.74 across all participants for 1–4 synergies, respectively (Figure 5). The average correlation coefficients were 0.79, 0.81, 0.81, and 0.78 for the TD children for 1–4 synergies, respectively; 0.79, 0.78, 0.79, and 0.74 for GMFCS Level I; 0.79, 0.76, 0.76, and 0.72 for GMFCS Level II; and 0.79, 0.75, 0.74, and 0.70 for GMFCS Level III. For an individual participant, the minimum correlation coefficient of synergy activations across LP cutoff frequencies was 0.51, 0.23, 0.40, and 0.45 for 1–4 synergies.

### Unit variance

**Figure 6:**
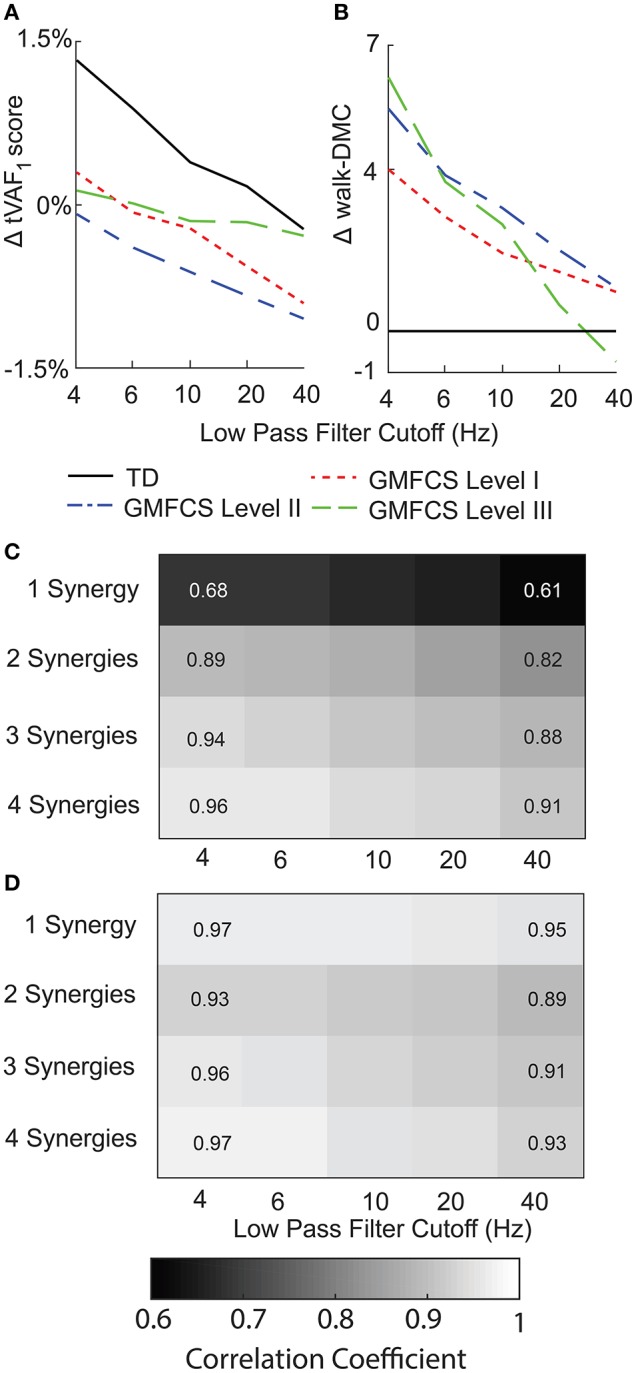
Average change in **(A)** tVAF and **(B)** walk-DMC for synergies calculated with EMG data scaled by peak activation or unit variance. Positive values indicate that results from unit variance scaling were greater than peak activation scaling. Average correlation coefficients of **(C)** synergy weights and **(D)** synergy activations between EMG data scaled by peak activation or unit variance.

Scaling EMG data to unit variance impacted synergy complexity, structure, and activations. Scaling to unit variance had a variable impact on tVAF_1_, increasing tVAF_1_ for some children and decreasing tVAF_1_ for others when compared to peak amplitude scaling (average difference across all participants: 1.7, SD 1.6 percentage points). However, group average tVAF_1_ changed only slightly with unit variance scaling, with a maximum change of 1.3 percentage points for TD with a 4 Hz LP filter (Figure 6A). Changes in walk-DMC due to unit variance scaling were largest with a 4 Hz LP filter with increases of 4.0, 5.4, and 6.2 points for GMFCS Levels I, II, and III and smallest with a 40 Hz LP with group changes of 1.0, 1.1, and -0.7 points, respectively (Figure 6B). The synergy weights correlation coefficients calculated with and without unit variance scaling were lowest for one synergy and decreased with greater LP cutoff frequency (Figure 6C). Synergy activations were similar between scaling methods and correlation coefficients slightly decreased with increasing LP filter cutoff (Figure 6D).

For EMG data scaled to unit variance, LP cutoff frequency caused slightly larger changes in tVAF_1_, with an average change of 10.5 percentage points between 4 Hz and 40 Hz LP filters. When we applied a threshold of tVAF > 90% to choose the number of synergies, 12% of children with CP and 11% of TD children had the same the number of synergies across all LP cutoff frequencies. Changes in walk-DMC were similar with changes of 1.2, 2.8, and 3.7 points for GMFCS Levels I, II, and III, respectively. The correlation coefficients of synergy weights were higher with unit variance scaling than EMG data normalized by peak amplitude, with average correlation coefficients of 0.90, 0.94, 0.95, and 0.96 for 1–4 synergies comparing 4 Hz and 40 Hz LP filters. The correlation coefficients of synergy activations were also slightly higher than EMG data normalized by peak amplitude, with average correlation coefficients of 0.81, 0.81, 0.79, and 0.77 for 1–4 synergies comparing 4 Hz and 40 Hz LP filters.

## Discussion

A z-score normalized measure of synergy complexity, walk-DMC, was more stable across LP filter parameters than tVAF or number of synergies. For both TD and CP children, tVAF decreased with increasing LP cutoff. Amplitude scaling of EMG data had smaller effects than LP filter choice on synergy complexity. These results highlight one disadvantage of using tVAF thresholds (i.e., tVAF > 90%) to identify the number of synergies for further analyses. Since tVAF is sensitive to filtering parameters, different studies may report different synergy numbers and co-activation patterns, depending on their choice of LP cutoff frequency. Despite the sensitivity of tVAF to LP cutoff, the TD and CP groups were significantly different across all LP filters for all measures of synergy complexity. These results suggest that z-score measures may be useful for comparing synergy results across studies or clinical centers. However, z-score normalization requires EMG data from TD or control participants, which may not be available at all institutions. Similarly, caution should be exercised when picking a single tVAF threshold for selecting number of synergies or comparing number of synergies across studies.

The choice of LP filter also affected individual muscle contributions (muscle weights) within each synergy. Synergy weights for solutions with fewer synergies (e.g., *n* = 2 synergies) were more sensitive to LP cutoff. Increasing the number of synergies increased similarity of synergy weights since fewer muscles were activated together in each synergy. In this study we used a clinical dataset with EMG data from five muscles. We anticipate that the impacts of LP filter choice on synergy weights may be greater for datasets that have more muscles, since more muscles would be activated together in each synergy (Steele et al., 2013). Our results support work by Chvatal and Ting (2012) who demonstrated that further smoothing EMG data LP filtered at 40 Hz by subsequently averaging across bins ranging from 10 to 200 ms resulted in similar synergy weights (similarity >0.85 for the selected number of synergies with a threshold of tVAF > 85%). The correlation coefficients of the synergy activation curves also decreased with increasing LP filter cutoff. The decrease in correlation with increasing LP cutoff for the activation curves is reflective of the input EMG data, which retains additional high frequency components when processed with a higher LP filter (Figure 2).

The choice of amplitude scaling between unit variance and peak amplitude also impacted the individual muscle weights within each synergy. Synergy weights and activations were more similar across LP filter conditions for EMG data scaled to unit variance than peak amplitude, since scaling by unit variance reduces differences between muscles that may impact synergy weights and activations. All amplitude scaling methods involve applying a unique scaling factor to each EMG channel, which impacts the scaling of calculated synergy weights. Note that scaling to unit variance negates the effects of any previous scaling (e.g., peak amplitude or maximum voluntary contraction). As with LP filter choice, analyses that calculated fewer synergies (e.g., *n* = 2 synergies) were more sensitive to amplitude scaling. The stronger influence of EMG processing methods on synergy solutions with fewer synergies is especially important for evaluations of clinical populations, which typically have reduced synergy complexity compared to control populations.

Just as we found a range of LP filters used in prior research, we also found a range of HP filters used before rectification, including 40 Hz (Bowden et al., 2010; Clark et al., 2010; Routson et al., 2014), 35 Hz (Torres-Oviedo and Ting, 2010; Sawers et al., 2015), and 20 Hz (Hug et al., 2012; Van der Krogt et al., 2016). We could not explore the effects of HP filter choice with our dataset since our data were originally recorded with an on-board 35 Hz HP filter. However, the International society of Electrophysiology and Kinesiology (ISEK) currently recommends a HP filter from 5 to 10 Hz (Merletti and Torino, 1999). De Luca et al. (2010) found that a 20 Hz HP filter was the best compromise between eliminating movement artifacts and retaining EMG power. HP filters primarily act to reduce DC drift in the EMG signal due to motion artifact and other nonphysiological signals. Consequently, we do not expect large impacts from HP filters on synergies, but the precise impacts of HP filtering on synergy analyses remain an open question.

Beyond filter cutoff frequency and amplitude scaling methods, there are other EMG preprocessing choices we did not explore, including filter type and filter order. Devaprakash et al. (2016) compared a 2nd order critically damped filter to a 2nd order Butterworth filter with consistent cutoff frequencies and found only small differences in the EMG data that did not affect clinical interpretation. (De Luca et al., 2010) found >1% difference in root mean square difference between EMG profiles processed with 2nd or 3rd order Butterworth filters. Taken together, these results suggest that filter choice and order are less significant than LP cutoff frequency for EMG and synergy analyses.

Given the wide variety of EMG data processing methods used in prior research, exploring and discussing the underlying biological mechanisms that should inform the choice of filters and synergy analyses methods would be useful for future research. Current EMG data processing methods are largely based upon technical specifications. Prior work has found that LP filters should be tailored to the specific task (Shiavi et al., 1998; Hug, 2011). However, there is a need to explore the neurophysiology underlying synergy analyses, especially considering some of the limitations of surface EMG data (Farina et al., 2004). For example, if synergies are driven by an underlying central pattern generator, what are the rates of these reflex loops, and how can these biological processes inform data preprocessing and interpretation of synergy analyses? If central pattern generators are driven by low-frequency mechanisms, then perhaps low-frequency LP cutoff frequencies are more appropriate. Future research, such as newly developed methods with direct central nervous system recordings and surface EMG data may assist in understanding these relationships (Godlove et al., 2016).

Output measures of synergy analyses including tVAF, synergies weights, and synergy activations were sensitive to EMG processing methods. We found that increasing LP filter cutoff frequency decreased synergy complexity, as measured by tVAF. Since tVAF is commonly used to identify the number of synergies, LP filter choice can impact conclusions about the number of synergies and muscle co-activation patterns from synergy analyses. Synergy weights and activations are less sensitive to LP cutoff frequency when calculated for two or more synergies. Future studies of synergy analyses and potential clinical applications should carefully consider and report EMG processing methods to enable comparisons across studies and institutions. As synergy analysis is adopted in clinical gait analysis to inform treatment planning, these results highlight the importance of carefully considering EMG processing methods and the utility of a control database. We found that z-score based measures, such as walk-DMC, that compare to control populations can reduce sensitivity to LP filter choice and facilitate comparisons between studies and clinical centers with different EMG protocols.

## Author contributions

MS provided the clinical EMG data. BS, MS and KS designed and crafted the research questions. BS wrote the code and analysed the data. BS, MS, and KS interpreted the results and drafted and revised the manuscript. BS, MS, KS assert that the contents of this research are their own and accurate as they have been presented.

### Conflict of interest statement

The authors declare that the research was conducted in the absence of any commercial or financial relationships that could be construed as a potential conflict of interest.
